# XELOX (capecitabine plus oxaliplatin) as first-line treatment for elderly patients over 70 years of age with advanced colorectal cancer

**DOI:** 10.1038/sj.bjc.6603047

**Published:** 2006-03-21

**Authors:** J Feliu, A Salud, P Escudero, L Lopez-Gómez, M Bolaños, A Galán, J-M Vicent, A Yubero, F Losa, J De Castro, M Á de Mon, E Casado, M González-Barón

**Affiliations:** 1 Service of Medical Oncology, H La Paz, Universidad Autónoma de Madrid, Paseo de la Castellana 261, Madrid 28046, Spain; 2 Service of Medical Oncology, H Arnau Vilanova, Avda. Alcalde Roure 80, Lérida 25006, Spain; 3 Service of Medical Oncology, H Lozano Blesa, C/San Juan Bosco 15, Zaragoza 50009, Spain; 4 Service of Medical Oncology, H Virgen de la Salud, Avda. Barber 35, Toledo 45004, Spain; 5 Service of Medical Oncology, H San Pedro de Alcántara Avda. Millán Astray s/n10003, Cáceres, Spain; 6 Service of Medical Oncology, H Sagunto, Avda. Ramón y Cajal s/n46520, Valencia, Spain; 7 Service of Medical Oncology, H General Universitario, Avda. Tres Cruces s/n46014, Valencia, Spain; 8 Service of Medical Oncology, H Obispo Polanco, Avda. Ruiz Jarabo s/n44002, Teruel, Spain; 9 Service of Medical Oncology, H de la Creu Roja, Avda. Josep Molins 29-41, Hospitalet de Llobregat, 08906 Barcelona, Spain; 10 Service of Medical Oncology, H Príncipe de Asturias, Ctra. Alcalá-Meco s/n Alcalá de Henares, 28880 Madrid, Spain

**Keywords:** capecitabine, oxaliplatin, XELOX, metastatic colorectal cancer, elderly

## Abstract

The purpose of this phase II trial was to determine the efficacy and safety of the XELOX (capecitabine/oxaliplatin) regimen as first-line therapy in the elderly patients with metastatic colorectal cancer (MCRC). A total of 50 patients with MCRC aged ⩾70 years received oxaliplatin 130 mg m^−2^ on day 1 followed by oral capecitabine 1000 mg m^−2^ twice daily on days 1–14 every 3 weeks. Patients with creatinine clearance 30–50 ml min^−1^ received a reduced dose of capecitabine (750 mg m^−2^ twice daily). By intent-to-treat analysis, the overall response rate was 36% (95% CI, 28–49%), with three (6%) complete and 15 (30%) partial responses. In total, 18 patients (36%) had stable disease and 14 (28%) progressed. The median times to disease progression and overall survival were 5.8 months (95% CI, 3.9–7.8 months) and 13.2 months (95% CI, 7.6–16.9 months), respectively. Capecitabine was well tolerated: grade 3/4 adverse events were observed in 14 (28%) patients: 11 (22%) diarrhoea, eight (16%) asthenia, seven (14%) nausea/vomiting, three (6%) neutropenia, three (6%) thrombocytopenia, and two (4%) hand–foot syndrome. There was one treatment-related death from diarrhoea and sepsis. In conclusion, XELOX is well tolerated in elderly patients, with respectable efficacy and a meaningful clinical benefit response. Given its ease of administration compared with combinations of oxaliplatin with 5-FU/LV, it represents a good therapeutic option in the elderly.

Colorectal cancer (CRC) is the second most common cause of death from cancer in industrialised countries (Jemal *et al*, 2003) and its incidence increases with age (Gatta *et al*, 1998). Because of a progressively ageing population, it can be expected that the number of elderly with CRC will increase significantly in coming decades. However, chemotherapy is used less frequently in the elderly than in other age groups, in both the adjuvant (Potosky *et al*, 2002) and metastatic (Simmonds and Best, 1999; Sundarajan *et al*, 1999) settings. Factors that influence the resistance to use chemotherapy in the elderly include: a general lack of studies in this age group; the fear that the progressive reduction of functional reserve that occurs in various organs with ageing might increase the susceptibility of the elderly to adverse effects that can reduce quality of life, particularly diarrhoea, mucositis, and myelosuppression (Balducci and Extermann, 2000) and comorbidity, which makes it difficult or impossible to use chemotherapy (De Marco *et al*, 2000).

Oxaliplatin and irinotecan are now established as first-line agents in the treatment of metastatic CRC (MCRC). The addition of oxaliplatin to 5-FU/leucovorin (LV) in first-line therapy has allowed an increase in response rates and time to disease progression (TTP) as compared to 5-FU/LV alone (de Gramont *et al*, 2000; Douillard *et al*, 2000; Giacchetti *et al*, 2000; Saltz *et al*, 2000; Grothey *et al*, 2002). In addition, it has been found recently that the combination of oxaliplatin with infusional 5-FU/LV significantly increases response rate, TTP, and overall survival as compared to the combination of irinotecan with bolus 5-FU/LV (Goldberg *et al*, 2004). These regimens have the inconvenience of being based on the requirement for continuous infusion, which necessitates permanent venous access and the need to carry infusion apparatus/fluids. This increases the risk of catheter-related complications, such as infections (Clark and Raffin, 1990) or thromboembolism (Prandoni and Bernardi, 1990; Verso and Agnelli, 2003).

Oral chemotherapy avoids the costs and inconveniences associated with continuous infusions and is preferred to i.v. therapy given equal efficacy (Liu *et al*, 1997; Borner *et al*, 2002b). Capecitabine (Xeloda®; Hoffmann-La Roche Inc., Nutley, NJ, USA) is an orally administered fluoropyrimidine designed to deliver 5-FU to tumour tissue (Miwa *et al*, 1998; Schüller *et al*, 2000). Large-scale, phase III trials have shown that capecitabine single-agent therapy is at least as effective and well tolerated as 5-FU/LV as first-line treatment for MCRC (Hoff *et al*, 2001; Van Cutsem *et al*, 2001) or as adjuvant therapy for stage III colon cancer (Scheithauer *et al*, 2003; Twelves *et al*, 2005). Furthermore, these trials showed that capecitabine had similar, favourable efficacy and safety in patients <65 or ⩾65 years. Various phase II/III studies have shown similar efficacy and safety for combination of oxaliplatin with either oral capecitabine or infusional 5-FU/LV (Borner *et al*, 2002a; Zeuli *et al*, 2003; Cassidy *et al*, 2004; Shields *et al*, 2004, Arkenau *et al*, 2005; Ducreux *et al* 2005; Sastre *et al*, 2005), although experience with these regimens in the elderly is very limited in these studies, and usually excluded those >75 years. In fact, the vast majority of trials of any chemotherapeutic agents in MCRC have not included trials of patients >70 years.

Given the characteristics of this combination with respect to efficacy, safety, and ease of administration, the use of capecitabine/oxaliplatin would be very attractive to use in the general population and particularly in the elderly in whom chemotherapy is usually restricted. Thus, the aim of our study was to analyse the efficacy and safety of the combination of capecitabine and oxaliplatin (XELOX) in patients ⩾70 years of age with advanced CRC.

## PATIENTS AND METHODS

### Patient population

Patients were required to be ambulatory and have an Eastern Cooperative Oncology Group (ECOG) performance status ⩽2, with a life expectancy of at least 3 months. All patients had measurable disease defined by the presence of at least one unidimensionally measurable lesion (RECIST criteria) (Therasse *et al*, 2000) by computed tomography scan. Pleural effusion, ascites, osteoblastic lesions, or previously irradiated lesions were not accepted as measurable disease.

Patients who had received prior adjuvant 5-FU-based chemotherapy were eligible if they had been disease free for at least 6 months after the completion of therapy. Patients who had received radiotherapy were eligible if there was at least one measurable lesion outside the radiation field. Other inclusion criteria were adequate haematological function (granulocyte count ⩾2 × 10^9^ l^−1^ and platelets >100 × 10^9^ l^−1^); adequate hepatic function (serum bilirubin <1.25 × the upper normal limit (UNL), glutamic oxaloacetic transaminase (SGOT), and glutamic pyruvic transaminases (SGPT) values <2.5 × UNL in the absence of hepatic metastases or <5 × UNL in the presence of metastasis); alkaline phosphatase <2.5 × UNL in the absence of hepatic metastases or < 5 × UNL or 10 × UNL in the presence of hepatic or bone metastases and adequate renal function (creatinine clearance ⩾30 ml min^−1^). All patients provided written informed consent according to local ethical committee directives.

Patients with operable metastatic disease were excluded from the study. Other exclusion criteria were as follows: clinically significant cardiac disease or myocardial infarction within the last 12 months; lack of physical integrity of the upper gastrointestinal tract or malabsorption syndrome; peripheral neuropathy of National Cancer Institute (NCI) grade 2 or greater; prior therapy with capecitabine, oxaliplatin, or irinotecan; known brain or meningeal metastases; and history of other malignancy, except basal cell carcinoma or adequately treated *in situ* cervical carcinoma.

Local research ethics committees approved the trial protocol and all patients provided written informed consent.

### Treatment plan

The study regimen consisted of oxaliplatin 130 mg m^−2^ as 120-min i.v. infusion on day 1 and oral capecitabine 1000 mg m^−2^ twice daily (2000 mg m^−2^ total daily dose) on days 1–14 every 3 weeks. In patients with a creatinine clearance of 30–50 ml min^−1^, the dosage of capecitabine was reduced to 750 mg m^−2^ twice daily (1500 mg m^−2^ total daily dose). The dose of capecitabine was rounded to the next closest dose that could be administered using a combination of 500- and 150-mg tablets. The two daily doses of capecitabine were administered 12±2 h apart, 30 min after meals (breakfast and evening meal), with approximately 200 ml of water.

The Cockcroft–Gault formula (Cockcroft and Gault, 1976) was used to calculate the creatinine clearance between cycles. If clearance was <30 ml min^−1^, treatment was interrupted. Cycles were repeated every 3 weeks for a minimum of three per patient, unless disease progressed. Patients with a partial response or stable disease continued chemotherapy until progression or the development of unacceptable adverse events. Patients could also continue capecitabine monotherapy after discontinuation of oxaliplatin because of toxicity.

Patients were evaluated for adverse events before each cycle and graded according to NCI Common Toxicity Criteria (version 2.0). For hand–foot syndrome, the previously published grading system was used (Blum *et al*, 1999). Dose reduction/interruption for capecitabine according to adverse event grade was performed as previously described (Blum *et al*, 1999). The oxaliplatin dose was reduced by 25% in the event of grade 3 or 4 thrombocytopenia, grade 4 neutropenia, or any other severe (⩾grade 3) organ toxicity, and for paresthesiae with pain or functional impairment >7 days, or paresthesiae with pain persistent between cycles. For paresthesiae with functional impairment persistent between cycles, oxaliplatin was discontinued. Treatment could be delayed for up to 2 weeks if symptomatic toxicity persisted, the absolute neutrophil count was lower than 1 × 10^9^ l^−1^, or the platelet count was lower than 75 × 10^9^ l^−1^. Subcutaneous administration of granulocyte colony-stimulating factor 5 *μ*g kg day^−1^ on 5 consecutive days was recommended in the former group of patients. Any patient who required more than 2 weeks for recovery from adverse reactions was excluded from the study.

### Pretreatment and follow-up evaluation

A diagnostic work-up was performed within 3 weeks prior to the start of treatment, consisting of a complete clinical history, physical examination, blood analysis (haematology and complete biochemistry), and imaging studies as needed (chest X-ray, computed tomography of the chest, abdomen and pelvis, abdominal ultrasound, and bone scan). The Charlson comorbidity scale (Charlson *et al*, 1987), Katz Activity of Daily Living (ADL) index (Katz *et al*, 1963), and Lawton Instrumental Activity of Daily Living (IADL) index (Lawton, 1998) were used in patient assessment. Patients' ECOG performance status and weight were also recorded. An ECG was performed in all patients prior to receiving study treatment. Symptom assessment, physical examination, and blood biochemistry were repeated before each treatment cycle. Tumour measurements were taken every three cycles or sooner if clinically indicated.

Patients and/or caregivers were given a card indicating the number of pills that they were to take per day: they were asked to record the dose actually taken and give the card back to the doctor at the next visit.

### Response criteria

Patients were evaluated clinically at least every 3 weeks and radiographically every 9 weeks. The same evaluation modality was used throughout the study. The RECIST response guidelines were used (Therasse *et al*, 2000), defining all responses after at least 9 weeks of therapy as follows: complete response (CR), partial response (PR), stable disease (SD), or progressive disease (PD). We defined disease control as the sum of patients achieving a CR, PR, or SD. Confirmation of all responses was required after 4 weeks. The TTP was estimated from the dates of the first course of treatment to the first documentation of disease progression. Survival was calculated by the same method from the date of the first cycle of treatment until the date of death or last known follow-up.

### Symptoms assessment

An assessment of clinical benefit was determined from a composite of pain intensity, analgesic consumption, ECOG performance status, weight loss, and the presence of anorexia and asthenia. The same doctor for each patient assessed ECOG performance status and symptoms before each chemotherapy cycle. Patients entered a pain stabilisation run-in period to establish baseline measures. Pain was assessed with the Memorial Pain Assessment Card (MPAC) (Fishman *et al*, 1987). Asthenia and anorexia were assessed using a visual analogue scale (VAS) of 0–100. In addition, patients' weight was measured at each visit. Patients were considered to be evaluable for palliative benefits when they initially had one of the following signs or symptoms: an ECOG performance status ⩾1; an MPAC score ⩾20; baseline analgesic consumption of ⩾10 morphine equivalents mg day^−1^; score on the VAS for anorexia and/or asthenia of ⩾20; or weight loss >10% during the previous 6 months.

Symptom improvement was defined as an improvement of at least one score from baseline in ECOG performance status; 5% weight gain from baseline (patients with oedema, ascites, or pleural effusion were excluded from this category); or an improvement of ⩾50% from baseline in disease-related symptoms (pain, use of analgesics, anorexia, and asthenia) and analgesic consumption (measured weekly in morphine-equivalent milligrams). Each of these improvements had to be sustained for at least 4 weeks (Cocconi *et al*, 1998).

### Statistical methods

The primary end point of the trial was to determine the activity of the XELOX regimen in the intent-to-treat population. Secondary objectives were the safety profile, clinical benefit, TTP, and overall survival. Dose intensity was calculated for each patient from the total dose of capecitabine and oxaliplatin administered during the entire course of treatment and expressed as the mean drug dose in mg m^−2^ week^−1^.

An optimal two-stage design as described by Simon (1989) was used. A true response probability of 40% or greater was considered to be of interest, while further testing of the regimen would not be pursued if the response probability was 20% or lower. In the first stage, a total of 13 patients were included and at least four responses (CR or PR) were required to continue to the second stage. In the second stage, 30 additional patients were included plus 10% to allow for losses. Univariate analysis was used to compare the rate of grade 3/4 adverse events according to age (70–79 years *vs* ⩾80 years), gender, creatinine clearance (<50 *vs* ⩾50 ml min^−1^), Charlson comorbidity scale (0 *vs* ⩾1), ECOG performance status (0 *vs* ⩾1), and ADL and IADL (independent *vs* dependent). Wilcoxon's signed-rank test (to compare quantitative variables) and Fisher's exact test (to compare percentages) were used. Overall survival and TTP were calculated using the Kaplan–Meier method.

## RESULTS

### Patient characteristics

From January 2003 to September 2003, 54 patients from 11 centres were included. Four patients were excluded from analysis because they did not meet all selection criteria. Their mean age was 76 (range, 70–82) years. The demographic and clinical characteristics of the patients are shown in Table 1, while Table 2 shows their functional assessments. One-third of patients were metastatic at the time of diagnosis, although only one-third had received prior adjuvant therapy. In five patients (10%), the primary tumour was not resected. The median time from the primary diagnosis of CRC to inclusion was 14.6 months. Regarding symptoms, 38 patients had significant symptoms at baseline evaluation, which included asthenia (56%), pain (40%), and anorexia (28%). In total, 28 patients (56%) had comorbid conditions, mainly hypertension, arthritis, obstructive lung disease, peptic ulcer disease, diabetes, coronary insufficiency, and cardiovascular conditions. Comorbidity was present in 20 (40% of) patients when using the Charlson scale (Charlson *et al*, 1987).

### Treatment duration

A total of 286 cycles of treatment were administered, with a median of five per patient (range 1–8 cycles). Eight (16%) patients received less than three cycles of XELOX, due to disease progression (*n*=2, 4%), because of adverse events (*n*=3, 6%), treatment-related death (*n*=1, 2%), lost to follow-up (*n*=1, 2%), and refusal to continue treatment (*n*=1, 2%). Nevertheless, all of the patients enrolled were considered evaluable for both efficacy and safety. A total of 23 (46%) patients experienced treatment delays during the study for causes unrelated to the treatment (*n*=10, 20%), haematological adverse events (*n*=5, 10%), and nonhaematological adverse events (*n*=8, 16%). The median dose intensity of capecitabine was higher for patients with creatinine clearance >50 ml min^−1^ (9.22 g m^−2^ week^−1^), and for the remaining 14 patients with creatinine clearance between 30 and 50 ml min^−1^ (7.68 g m^−2^ week^−1^). These doses correspond to 86 and 98% of the predicted dose intensity, respectively. The median dose intensity of oxaliplatin was 39.8 mg m^−2^ week^−1^, which corresponded to 92% of the predicted dose intensity. During the course of their disease, 12 patients (24%) needed a central venous access: two during the XELOX treatment, six during the second-line treatment, and the rest of the patients after completion of chemotherapy for symptomatic treatment purposes.

### Efficacy

The assessment of response was performed on the ITT population of 50 eligible patients. Of these, three patients (6% of the eligible population) had a CR and 15 (30%) a PR. A total of 18 patients (36%) maintained SD and 14 (28%) had PD. The overall response rate was 36% (95% confidence interval (CI), 23–49%). There was no relationship between the rate of response and the location of metastases, ECOG performance status, ADL, IADL, or patients' age.

**Figure 1:**
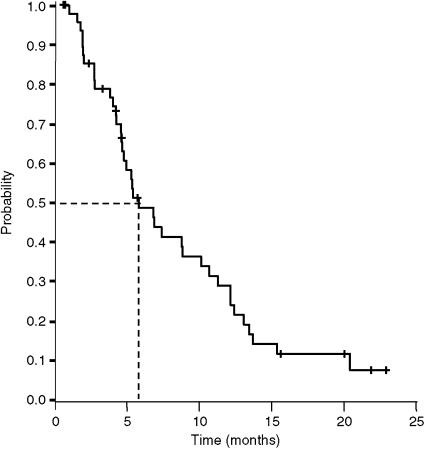
Time to disease progression (*n*=50).

**Figure 2:**
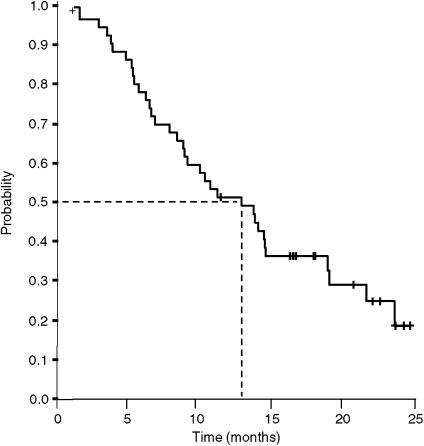
Overall survival (*n*=50).

The median TTP was 5.8 months (95% CI, 3.9–7.8 months; Figure 1), median overall survival was 13.2 months (95% CI, 7.6–16.9 months; Figure 2), and the actuarial survival per year was 51% (95% CI, 37–65%). Second-line chemotherapy based on irinotecan was subsequently administered in 12 patients (24%).

Of the 50 enrolled patients, 38 were evaluable for symptoms or ECOG score response. The remaining 12 patients were not evaluable for the following reasons: ECOG=0, MPAC score <20, or VAS for anorexia and asthenia <20. Clinical benefit responses are summarised in Table 3. Overall, 16 patients (42%; 95% CI, 26.7–59.6%) obtained an improvement in ECOG score and/or at least one symptom without worsening in other symptoms or ECOG score. The median duration of this improvement was 5.3 months (95% CI, 2–12 months).

### Safety

**Figure 3:**
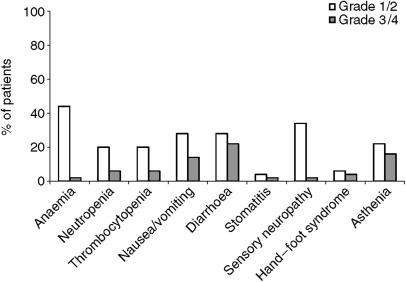
Adverse events by NCI-CTC grade (*n*=50).

Treatment was generally well tolerated. In all, 32 patients (64%) had an adverse event, usually grade 1/2. The main adverse events were gastrointestinal and haematological (Figure 3). A total of 14 patients (28%) had grade 3/4 adverse events: 11 (22%) diarrhoea, eight (16%) asthenia, seven (14%) nausea/vomiting, three (6%) neutropenia, three (6%) thrombocytopenia, two (4%) hand–foot syndrome, and one each (2%) with anaemia, mucositis, and sensory neuropathy, respectively. In a patient with a protocol violation, there was one treatment-related death due to diarrhoea and sepsis: the dose of capecitabine was not reduced to 75% due to creatinine clearance at 38 ml min^−1^.

An exploratory univariate analysis did not detect any relationships between the appearance of grade 3/4 adverse events and age (70–79 years *vs* ⩾79 years), gender, ADL, IADL, ECOG status, comorbidity, or renal function.

## DISCUSSION

Currently, MCRC is an incurable disease. Thus, treatment must be directed at survival prolongation, symptom alleviation, and improving, or at least maintaining, the patient's quality of life. These objectives can be achieved using the administration of less toxic chemotherapy regimens that are easier to administer and do not require the patient to be hospitalised repeatedly. With the elderly, we have to be even more careful in the choice of treatment. It is necessary to consider other aspects that might influence tolerability, such as the pharmacokinetic and pharmacodynamic changes that appear with age (Lichtman, 2004) and comorbidity (Balducci and Extermann, 2000).

The aim of our study was to analyse the efficacy and safety of the XELOX regimen in elderly patients with MCRC. Our results, with a response rate of 36% and a median TTP of 5.8 months, are similar to those obtained by other authors with capecitabine/oxaliplatin (Borner *et al*, 2002a; Zeuli *et al*, 2003; Cassidy *et al*, 2004) in series that excluded patients aged >75 years. In contrast, median overall survival in our series was 13.2 months, which is somewhat less than the 17–20 months reached in these other studies (Borner *et al*, 2002a; Zeuli *et al*, 2003; Cassidy *et al*, 2004). However, median overall survival was similar to that obtained in studies performed exclusively in the elderly with MCRC who received combinations of 5-FU/LV plus oxaliplatin or irinotecan (Aparicio *et al*, 2003), or capecitabine plus oxaliplatin (Comella *et al*, 2005). It has been noted that the survival of patients with MCRC increases when they receive three active cytostatic agents (5-FU/LV, oxaliplatin, and irinotecan) during their disease course (Grothey *et al*, 2004; Tournigand *et al*, 2004). Thus, the shorter survival of the patients in our series might be attributable to the low proportion of elderly who received a second line of chemotherapy. For example, it is clear from this study that irinotecan was not a widely used second line. The frequent comorbidity that is often present might also contribute towards this. It would therefore appear necessary to increase the proportion of elderly to whom second-line treatment has been added after disease progression, especially if there is little comorbidity, or little or no ADL/IADL dependence. It is possible that the increasing availability of new targeted agents could increase the efficacy of treatment in elderly patients.

The XELOX regimen was generally well tolerated. In total, 28% of the patients experienced grade 3/4 adverse events, which is comparable to the safety described with this capecitabine/oxaliplatin in other series that included patients who were not elderly ((Borner *et al*, 2002a; Zeuli *et al*, 2003; Cassidy *et al*, 2004; Shields *et al*, 2004). The low rate of grade 3/4 neurotoxicity in our patients might appear somewhat surprising at only 2%, especially compared with the 17% reported by Cassidy *et al* (2004). However, given that the development of this toxicity depends on the total accumulated dose of oxaliplatin received during treatment, the low toxicity in our series can probably be attributed to the reduced number of treatment cycles received by our patients compared to the series of Cassidy *et al* (2004) (median 4.5 *vs* 8 cycles, respectively). On the other hand, the proportion of the elderly in our study that developed grade 3/4 adverse events was similar to the 29% reported in another study performed exclusively in the elderly and which also used a capecitabine/oxaliplatin regimen, in which the dose of subsequent cycles was increased as a function of tolerability by the patient (Comella *et al*, 2005).

The good tolerability of the XELOX regimen in our series might, in part, be due to the dose of capecitabine used, which was adjusted for each cycle as a function of creatinine clearance (Gieschke *et al*, 2003; Feliu *et al*, 2005). We believe that this is important in the elderly. Moreover, the only toxic death in our study occurred in a patient where the dose was not appropriately adjusted because of his low creatinine clearance. The safety of XELOX was clearly better than when using combinations of 5-FU/LV plus oxaliplatin in the elderly with MCRC: grade 3/4 adverse events occurred in 28% of patients in our series compared to 42–80% in series that received 5-FU/LV/oxaliplatin (Aparicio *et al*, 2003; Figer *et al*, 2004). Of particular note is the high rate of grade 3/4 neutropenia that is seen with 5-FU/LV/oxaliplatin regimens, which varies in the elderly between 30 and 55% according to the series considered (Giacchetti *et al*, 2000; Baretta *et al*, 2001; Aparicio *et al*, 2003; Mattioli *et al*, 2003; Figer *et al*, 2004), whereas this adverse event was detected in <10% of patients with capecitabine/oxaliplatin (Borner *et al*, 2002a; Zeuli *et al*, 2003; Cassidy *et al*, 2004; Shields *et al*, 2004; Arkenau *et al*, 2005). Additionally in our study, we did not encounter any relationship between toxicity and certain factors that are generally presumed to be capable of predisposing to greater toxicity, such as female gender (Macdonald, 2002) comorbidity (Balducci and Extermann, 2000), performance status, etc. However, we acknowledge that the small number of patients means that our study might be insufficiently powered to detect these relationships.

In addition to the potential efficacy and safety advantages of replacing 5-FU with capecitabine, it is important to consider the ease of oral administration, which avoids the possible complications and inconveniences associated with the use of infusion apparatus or hospital admissions (Clark and Raffin, 1990; Prandoni and Bernardi, 1990; Verso and Agnelli, 2003). Also, the number of hospital visits can be reduced considerably to once every 3 weeks with XELOX, when the patient is routinely reviewed, oxaliplatin administered, and capecitabine dispensed. This might prove especially interesting in elderly patients, who usually require the assistance of a family member or helper to get to hospital. It is still important for patients to receive good quality educational materials and to have regular phone contact with their nurse/physician. Patients with dependency for the daily activities of life may still require a caregiver who takes full responsibility of administering treatment and the early detection of adverse events. Furthermore, the addition of targeted therapy with bevacizumab can be conveniently integrated into the 3-weekly XELOX regimen with little impact on regimen convenience.

In summary, the results of our study show that the XELOX regimen represents a good therapeutic option in the fit elderly and in the patient partially dependent and with little severe comorbidity. In addition, it appears safer than that previously reported in studies with 5-FU/LV plus oxaliplatin, especially in the elderly. While we do not have available the results of phase III studies in the elderly comparing the efficacy and tolerability of the XELOX regimen *vs* 5-FU/LV plus oxaliplatin, its convenience and ease of administration lead us to conclude that XELOX might be a good combination.

## Figures and Tables

**Table 1:** Patient characteristics at study entry (*n*=50)

**Characteristic**	**Patients (%)**
*Age (years)*	
70–75	56
76–79	32
⩾80	12
	
*Gender*	
Male	72
Female	28
	
*ECOG performance status*	
0	52
1	46
2	2
	
*Location of primary tumour*	
Colon	68
Rectum	28
Both	4
	
*No. of metastatic sites*	
1	74
⩾2	26
	
*Differentiation*	
Well differentiated	10
Moderately differentiated	60
Poorly differentiated	18
Undetermined/unknown	12
	
*Sites of metastasis*	
Liver	64
Lung	32
Lymph node	14
Other	24

ECOG=Eastern Cooperative Oncology Group.

**Table 2:** Functional assessments at entry (*n*=50)

**Functional assessment**	**Patients (%)**
*Activity of daily living index*	
No dependence	58
Light dependency	24
Moderate dependency	14
Serious dependency	4
	
*Instrumental activity of daily living index*	
Autonomous	58
Light dependency	30
Moderate dependency	8
Serious dependency	4
	
*Charlson comorbidity scale*	
0	60
1	26
2	8
3	6

**Table 3:** Effect of the treatment on performance status and symptom (*n*=38)

	**Patients (%)**		
**Parameter**	**Improvement**	**Stabilisation**	**Worsening**
ECOG performance status	20	60	20
Pain	23	63	14
Anorexia	26	54	20
Asthenia	37	37	26
Weight loss	26	60	14

ECOG=Eastern Cooperative Oncology Group.
